# Characterization of the complete chloroplast genome of *Asparagus filicinus* (Asparagaceae: Asparagoideae: *Asparagus*), a traditional Tibetan medicinal plant

**DOI:** 10.1080/23802359.2019.1666688

**Published:** 2019-09-20

**Authors:** Qien Li, Xianjia Li, Duojie, Dongzhi Duojie, Banma Cairen, Xiao Guo

**Affiliations:** a Tibetan Medicine Research Center of Qinghai University, Qinghai University Tibetan Medical College, Xining, Qinghai, People’s Republic of China;; b State Key Laboratory of Tibetan Medicine Research and Development, Qinghai Tibetan Medicine Research Institute, Xining, Qinghai, People’s Republic of China

**Keywords:** complete chloroplast genome, *Asparagus filicinus*, genome assembly, phylogeny

## Abstract

*Asparagus filicinus* is a traditional medicinal plant with the treatment of pneumonia and cancer, which has been classified as threatened due to habitat destruction and over-harvesting. In this study, its complete chloroplast genome was assembled from the whole genome Illumina sequencing data. The circular genome was 156,674 bp long, containing a large single copy (LSC) region of 85,003 bp and a small single copy (SSC) region of 18,663 bp, which were separated by a pair of 26,504 bp inverted repeat (IR) regions. It encoded a total of 126 genes, including 72 protein-coding genes, 46 tRNA genes and eight rRNA genes. The most of gene species occurred as a single copy, while 17 gene species occurred in double copies. The overall A + T content was 62.4%, while the corresponding values of the LSC, SSC and IR regions were 64.5, 68.4 and 57.1%, respectively. Phylogenetic analysis indicated that *A*. *filicinus* was relatively close to two species belonging to the subgenus *Asparagus*.

*Asparagus filicinus* Ham. ex D. Don. is a traditional medicinal plant of the Asparagoideae within the family Asparagaceae, which is distributed discontinuously in the northern and south-western China, as well as in Bhutan, India, Myanmar and Thailand (Tang and Wang 1978). The dried root of this plant has been reported for its usage as antipyretic, antitussive, diuretic and expectorant. Steroidal saponins are generally considered pharmacologically important for the treatment of pneumonia and cancer (Zhou et al. 2007). However, it has been classified as threatened in the IUCN Red List of Threatened Species since 2016 (IUCN 2017), mainly because of its sharp decrease of population size due to habitat destruction and over-harvesting. In addition, Fukuda et al. (2005) investigate the phylogenetic relationships within the genus *Asparagus*, which suggested that *A*. *filicinus* is one species of the subgenus *Asparagus* and showed very close relationship with another species *A*. *schoberioides*. However, the problem of its taxonomical phylogenetical position is still doubtful due to lack of more genomic information. In the present study, the complete chloroplast genome sequence of *A*. *filicinus* is reported for contributing to conservation of this species, and also providing significant information for its phylogenetic placement within the family Asparagaceae.

Genomic DNA was isolated from fresh leaves of an individual of *A*. *filicinus* collected from the Qinling Mountain (108°26′49″E, 35°26′37″N; the specimen was deposited at Qinghai University; accession number: LQE-2017-124). The whole-genome sequencing was conducted on an Illumina Hiseq X Ten platform. The resultant clean reads were then assembled into complete chloroplast genome with the program Velvet (Zerbino and Birney 2008), with *A*. *officinalis* (GenBank: LN896356.1) as the starting reference. The complete chloroplast genome was annotated using Geneious (Kearse et al. 2012) and then submitted to GenBank (accession no. MK920078).

The complete chloroplast genome of the *A*. *filicinus* was 156,674 bp in size. It possessed a typical quadripartite structure with two identical copies of a large inverted repeat separated by large and small single copy regions. The large single copy region (LSC) in *A*. *filicinus* was 85,003 bp and the small single copy region (SSC) was 18,663 bp, which were separated by a pair of 26,504 bp inverted repeat regions (IRs). The circular genome contained 126 genes, including 72 protein-coding genes (66 PCG species), eight ribosomal RNA genes (4 rRNA species) and 46 tRNA genes (31tRNA species). The most of gene species occurred in a single copy, while 17 gene species occurred in double copies, including four rRNA species (4.5S, 5S, 16S and 23S rRNA), 7 tRNA species and 6 PCG species. The overall A + T content of the circular genome was 62.4%, while the corresponding values of the LSC, SSC and IR regions were 64.5, 68.4and 57.1%, respectively.

A neighbor-joining tree (Figure 1) was reconstructed based on the complete chloroplast genome sequences of *A*. *filicinus* and other 26 species from Asparagaceae with the program MEGA6 (Tamura et al. 2013). The phylogenetic analysis supported the traditional taxonomy of the family Apocynaceae at the subfamily level, as well as at the genus level. *A*. *filicinus* was found to be relatively closely related to two species (*Asparagus* *officinalis* and *A*. *schoberioides*) from the same subgenus, which was consistent with the phylogenic study suggested by Fukuda et al. (2005).

**Figure 1.**
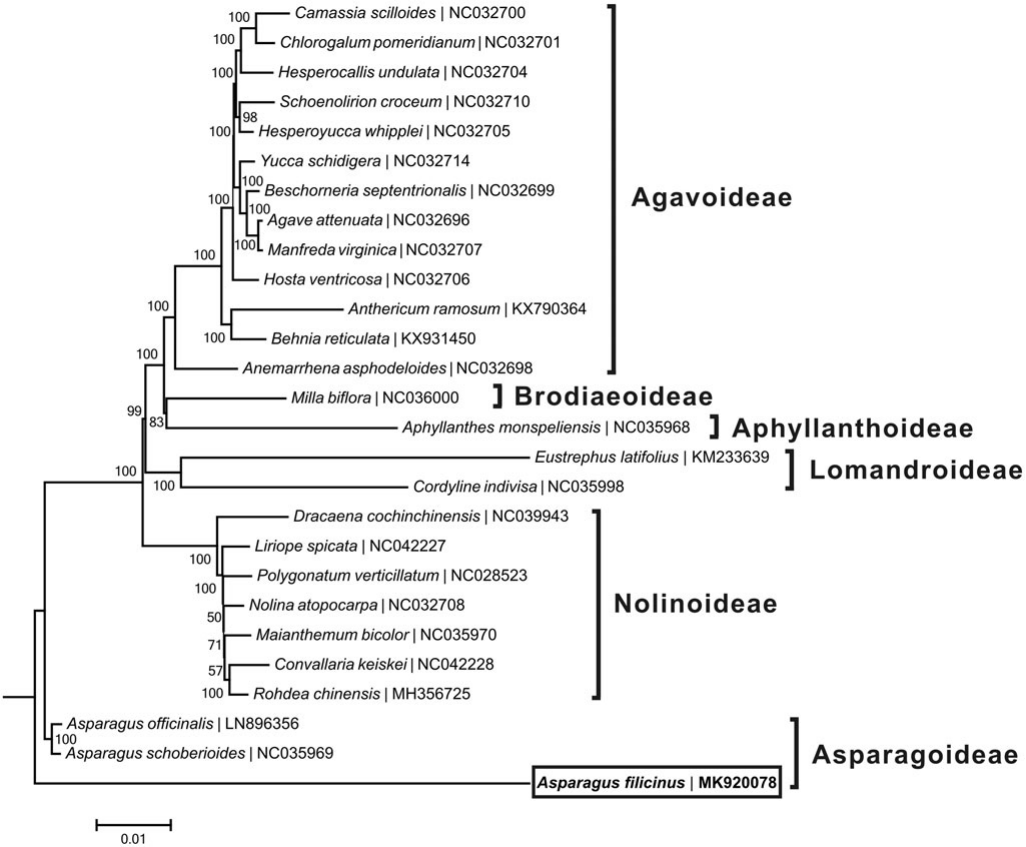
Neighbor-joining tree based on the complete chloroplast genome sequences of *A. filicinus* and related taxa within the family Asparagaceae. The numbers on the branches are bootstrap values. The accession number of GenBank for each species is list in figure.

## References

[CIT0001] Fukuda T, Ashizawa H, Suzuki R, Ochiai T, Nakamura T, Kanno A, Kameya T, Yokoyama J 2005 Molecular phylogeny of the genus *Asparagus* (Asparagaceae) inferred from plastid petb intron and petd–rpoa intergenic spacer sequences. Plant Spec Biol. 20:121–132.

[CIT0002] [IUCN] International Union for Conservation of Nature and Natural Resources. 2017 The IUCN red list of threatened species. [accessed 29 November 2017]. http://www.iucnredlist.org

[CIT0003] Kearse M, Moir R, Wilson A, Stones-Havas S, Cheung M, Sturrock S, Buxton S, Cooper A, Markowitz S, Duran C, et al. 2012 Geneious Basic: an integrated and extendable desktop software platform for the organization and analysis of sequence data. Bioinformatics. 28:1647–1649. 22543367 10.1093/bioinformatics/bts199 PMC3371832

[CIT0004] Tamura K, Stecher G, Peterson D, Filipski A, Kumar S 2013 MEGA6: molecular evolutionary genetics analysis version 6.0. Mol Biol Evol. 30:2725–2729. 24132122 10.1093/molbev/mst197 PMC3840312

[CIT0005] Tang J, Wang FZ 1978 Liliaceae In: Wu ZY, editors. Flora of China. Vol. 15 Beijing: Science Press; p. 104–106.

[CIT0006] Zerbino DR, Birney E 2008 Velvet: algorithms for de novo short read assembly using de Bruijn graphs. Genome Res. 18:821–829. 18349386 10.1101/gr.074492.107 PMC2336801

[CIT0007] Zhou LB, Chen TH, Bastow KF, Shibano M, Lee KH, Chen DF 2007 Filiasparosides A-D, cytotoxic steroidal saponins from the roots of *Asparagus filicinus*. J Nat Prod. 70:1263–1267. 17629328 10.1021/np070138w

